# External root resorption evaluated by CBCT 3D models superimposition

**DOI:** 10.1590/2177-6709.27.2.e2219315.oar

**Published:** 2022-06-10

**Authors:** Ana Beatriz N. PEREIRA, Rhita ALMEIDA, Flavia ARTESE, Camila DARDENGO, Cátia QUINTÃO, Felipe CARVALHO

**Affiliations:** 1 Universidade do Estado do Rio de Janeiro, Departamento de Ortodontia (Rio de Janeiro/RJ, Brazil).

**Keywords:** Root resorption, Orthodontic tooth movement, X-ray computed tomography

## Abstract

**Introduction:**

The literature reports the association of external root resorption (ERR) with orthodontic movement. In cases of premolars extractions, orthodontic movement of anterior teeth is usually quite expressive, which are precisely the most susceptible teeth to suffer from ERR.

**Objective:**

The aim of this study was to assess the root morphology of maxillary canines and incisors in patients submitted to four premolar extraction and orthodontic retraction of the anterior teeth, by means of 3D surface models superimposition and mapping.

**Methods:**

The sample consisted of six adult patients, five female and one male, with a mean age of 23.5 ± 6.5 years, who underwent orthodontic treatment. All patients presented bimaxillary dental protrusion, with indication of maxillary and mandibular first premolar extractions, followed by the retraction of anterior teeth and space closure. Cone beam CT scans were performed before the beginning of the treatment (T_0_) and right after space closure (T_1_). 3D models were built at both times and superimposed to identify the root changes for the given period.

**Results:**

All average differences were close to zero and, even when evaluating the extreme values, the observed changes were always smaller than the accuracy of the CBCT.

**Conclusion:**

A mild resorption trend was observed, although it was not clinically significant, with values lower than the tomography accuracy.

## RESUMO

**Introdução:**

A literatura relata a associação de reabsorção radicular externa (RRE) com a movimentação ortodôntica. Nos casos de extrações de pré-molares, a movimentação ortodôntica costuma ser bastante expressiva nos dentes anteriores, que são justamente os dentes mais suscetíveis à RRE.

**Objetivo:**

O objetivo do presente estudo foi avaliar a morfologia radicular de caninos e incisivos superiores em pacientes submetidos à extração de quatro pré-molares e retração ortodôntica dos dentes anteriores, por meio da sobreposição e mapeamento de modelos 3D.

**Métodos:**

A amostra foi composta por seis pacientes adultos, cinco do sexo feminino e um do masculino, com média de idade de 23,5 ± 6,5 anos, submetidos a tratamento ortodôntico. Todos os pacientes apresentavam biprotrusão dentária, com indicação de extração dos primeiros pré-molares superior e inferior, seguida de retração dos dentes anteriores e fechamento do espaço. A tomografia computadorizada de feixe cônico (TCFC) foi realizada antes do início do tratamento (T_0_) e logo após o fechamento de espaços (T_1_). Modelos 3D foram construídos em ambos os momentos e sobrepostos para identificar as mudanças nas raízes dentárias no período determinado.

**Resultados:**

Todas as médias das diferenças foram próximas de zero e, mesmo avaliando os valores extremos, as mudanças observadas foram sempre menores que a sensibilidade da TCFC.

**Conclusão:**

Observou-se tendência de leve reabsorção, embora não clinicamente significativa, com valores inferiores à sensibilidade tomográfica.

## INTRODUCTION

External root resorption (ERR) is characterized by the permanent shortening of the tooth root, which is a common clinical complication of orthodontic treatment. Although ERR may occur in any or all teeth, it most often involves the maxillary incisors.^1^ ERR is a sterile inflammatory process that is extremely complex and involves various components such as forces, tooth roots, bone, cells, surrounding matrix, and certain known biologic messengers.^2^

ERR is undesirable because it can affect the long-term viability of the dentition. The etiologic factors are complex and multifactorial, including individual biologic variability, genetic predisposition, and the effect of mechanical factors.^3^ ERR can also depend on the orthodontic technique, tooth and jaw morphologies, and presence of root resorption before treatment.^4^ Some general dentists believe that ERR is avoidable and blame orthodontists when it occurs during orthodontic treatment.^5^ The patient/parents must be informed about the risk of root resorption as a consequence of orthodontic treatment. For precaution, after six months of treatment, periapical radiographs of the teeth should be obtained and, when ERR is detected, treatment should be halted for two to three months, with passive archwires.^2^ After treatment, if severe ERR is shown on the final radiographs, follow-up radiographic examinations can be recommended until the resorption has been stabilized, which usually occurs after appliance removal. If it continues, sequential endodontic treatment with calcium hydroxide may be considered^6^, although this treatment is controversial.^7^

The use of conventional two-dimensional (2D) radiograph is not accurate for the detection of mild resorption.^8^ Furthermore, 2D radiograph may not represent the resorption lesions and their dimensions, which depend on the severity of the root resorption.^9^ ^,^ ^10^ Clinically, radiographs are an important diagnostic tool in detecting ERR, but the varying degrees of magnification and the limitation of 2D measurement of a 3-dimensional phenomenon make the quantitative value of radiographs questionable and geometrically inaccurate.^11^ ^,^ ^12^ Savoldi et al.^13^ have proposed a trigonometric correction for the use of panoramic radiographs to try to overcome these issues, but it still presents the limitations of a two-dimensional exam.

Recent studies suggest that CBCT is a more sensitive imaging modality for detecting root resorption than conventional radiograph.^14^ However, further studies are needed to assess the safety and cost effectiveness of CBCT in the management of orthodontic patients with ERR.^9^ ^,^ ^15^

Various three-dimensional (3D) superimposition methods are used for clinical diagnosis and treatment evaluation purposes in orthodontic treatment and craniofacial surgeries, but each method has valuable benefits and some limitations.^16^ CBCT images provide both crown and root information, which makes it possible to reconstruct a complete tooth model. With the digital model, orthodontists perform diagnosis and treatment planning through manipulating the tooth model in a graphical user interface, thereby realizing digital, efficient, and accurate orthodontic treatment.^17^ For these factors, CBCT was the imaging method chosen in this study, and segmentation of 3D models would be useful to allow the mapping, localization and quantification of root resorption in 3D virtual models.

Thus, the aim of this study was to assess the root morphology of maxillary canines and incisors in patients submitted to four premolar extraction and orthodontic retraction of the anterior teeth, by means of 3D surface models superimposition and mapping.

## MATERIAL AND METHODS

This preliminary prospective study, of experimental character, evaluated six adult patients, five female and one male, with a mean age of 23.5 ± 6.5 years, who underwent orthodontic treatment at the orthodontic clinic of *Universidade do Estado do Rio de Janeiro* (Rio de Janeiro/RJ, Brazil). All selected patients signed an informed consent form, and the experimental protocol was approved by the Institutional Review Board of the aforementioned university. 

The inclusion criteria of the sample were as follows: Angle Class I malocclusion; absence of vertical and transverse occlusal problems; bimaxillary protrusion (measured by interincisal angle <131º); mild dental crowding (up to 4mm); convex profile; presence of all teeth (except third molars); indication of orthodontic treatment with four first premolars extraction (IMPA > 87º); and good general health. The exclusion criteria were unavailability of time to attend appointment; severe systemic or psychological illness; active periodontal disease; parafunction, reflux or eating disorders; and missing teeth.

All selected patients (n = 6) were treated with the extraction of four first premolars, and space closure was carried out by an *en masse* retraction, or by two-stage closure technique. Morelli^®^ brackets (Sorocaba/SP, Brazil) were used for the standard Edgewise technique, with 0.022 x 0.028-in slots. Initial alignment was done without the inclusion of second molars, and the archwire sequence was the same for all patients, beginning with a 0.014-in nickel-titanium (NiTi), followed by 0.016-in and 0.018-in stainless steel (SS). After the extraction of the first premolars, the space closure was done as described above. All orthodontic activations were performed by the same clinician.

The incisors retraction was carried out using a 0.019 x 0.025-in rectangular SS archwire, with a 7-mm drop loop located distal to incisors or canines, without the inclusion of the second molars. Activation was standardized by opening the loops 1mm. After the complete space closure, a new CBCT (T_1_) was acquired. All patients in the sample received a comprehensive orthodontic treatment regardless of the time needed for the finishing period.

CBCT was taken with an i-CAT, Classic model (Imaging Sciences International, Hatfield, PA, USA). The field of view (FOV) was set to 16 x 13cm, the exposure time was 20 seconds, and the images were generated with isometric voxels of 0.3mm. The T_0_ and T_1_ scans of the six evaluated patients were exported to the Digital Imaging and Communications in Medicine (DICOM) format.

**Figure 1:**
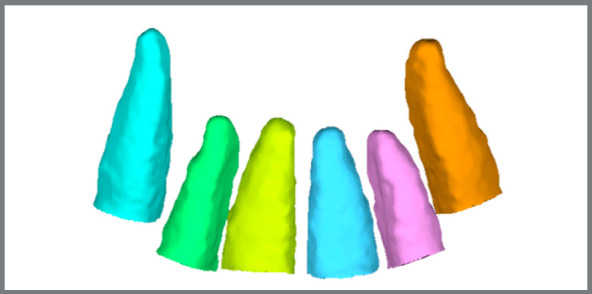
Example of 3D models in. STL format: root segmentations of the teeth #11, #12, #13, #21, #22 and #23 after superposition in the two different time periods.

**Figure 2:**
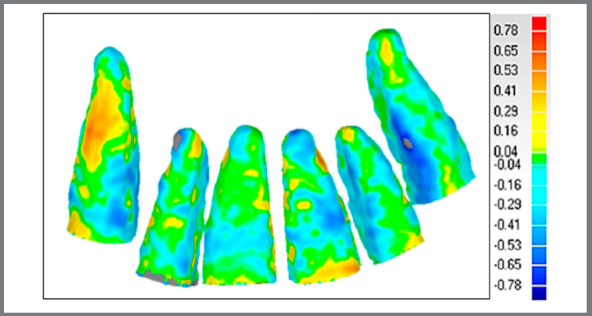
Example of colored map obtained: Quantification of changes between two segmentations of the same teeth, in mm. The map shows a resorption tendency, since the blue color is more predominant than the red.

**Figure 3:**
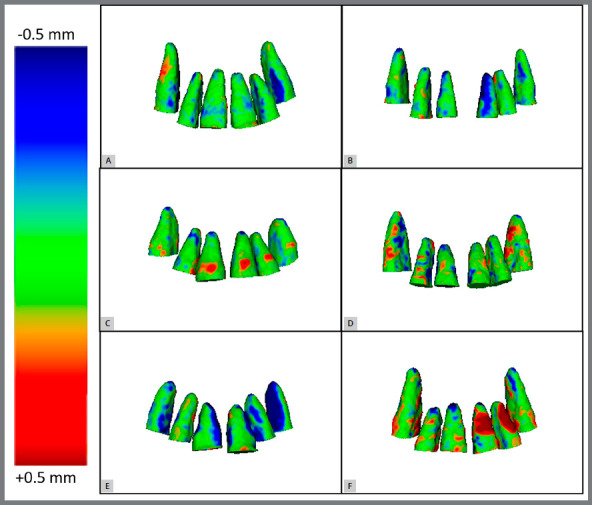
Qualitative result represented by colored maps, showing the difference between the two time periods of each patient, in a front view: A) Patient 1; B) Patient 2; C) Patient 3; D) Patient 4; E) Patient 5; F) Patient 6.

Afterwards, the CBCT postprocessing was as follows:

(1) The 3D models of anatomical structures of interest (dental elements: #13, #12, #11, #21, #22 and #23) were built with the aid of the open source software ITK-SNAP 3.6^18^, which uses a semi-automatic segmentation method (Fig 1).
(2) Each tooth was individually exported to a STL (Standard Triangulation Language) file.
(3) All models of T_0_ and T_1_ teeth were imported by the Geomagic Qualify 2013^®^ software (Geomagic U.S. Corp. Research Triangle Park, NC, USA).
(4) The T_0_ was considered as reference (fixed model), and the T_1_ was superimposed to the T_0_ using a best-fit alignment algorithm. As the purpose of the superimposition was to evaluate the root changes, only the coronal portion of the teeth above the base of the bracket was considered for the best-fit alignment (Fig 2).
(5) At this stage, all dental crowns were excluded. This portion of the teeth was selected considering the cementoenamel junction (CEJ) as the upper limit, and the lower limit comprised a plane defined by three points: (a) most superior mesial edge of the bracket; (b) most superior distal edge of the bracket, and (c) most prominent point of the cingulum. This area was used as a reference since the crown keep its shape across treatment, and the exclusion of the bracket was carried out because of the difficulty to consistently segment it across different timepoints, due to the artifacts generated by metal to the CBCT. The landmark placement of the upper and lower limits was defined after the initial whole tooth best-fit alignment, which exactly assures the same limit for T_0_ and T_1_.
(6) The qualitative comparison between the time points, comprising each tooth category separately, was done with the aid of color coded maps (Fig 3). Outward remodeling is represented by moving the scale towards the red color, while inward changes are represented by moving towards the blue color in the scale. The green color represents the absence of changes.
(7) Quantitative comparison between the superimposed surfaces used the root mean square (RMS) value, which represents the mean changes regardless of being inwards or outwards on the whole evaluated surface. The individual descriptive statistics of each case were computed (maximum and minimum distances between the surfaces, mean distance between the points of the two surfaces, standard deviation of distances, and mean square error of distances) (Table 1).

**Table 1:** Descriptive statistics for each tooth.

Tooth	#13	#12	#11	#21	#22	#23
Mean	-0.04	-0.06	-0.08	-0.04	-0.04	-0.14
Standard- deviation	0.09	0.08	0.08	0.08	0.09	0.29
Maximum	0.09	0.04	0.01	0.07	0.13	0.12
Minimum	-0.16	-0.2	- 0.17	-0.15	-0.16	-0.70
*p* (one sample *t*-test)	0.34	0.14	0.06	0,34	0.39	0.28
RMS*	0.17	0.19	0.23	0.18	0.19	0.21

* root mean square.

## STATISTICAL ANALYSIS

In this study, using a significance level of 0.05, a power level of 90%, a standard-deviation over patients of 0.08, and the detection of differences up to 0.7mm, the sample calculation determined the need of three patients (n = 3) for the sample. The “n” of this study was six, which surpasses the minimum verified for the conditions defined by the sample calculation. The Statistical Package for Social Sciences 22.0 (SPSS Inc., Chicago, IL, USA) was used for data analysis. The normality of the sample was verified by the Shapiro-Wilk test (Table 2). The Levene test verified the variance homogeneity (*p* = 0.405). To evaluate whether the difference between T_0_ and T_1_ was statistically different from zero, the one-sample *t*-test was used. The alpha level considered for all analyzes was 0.05. The evaluation of each tooth was carried out as independent subjects, so it was possible to observe how specific anatomical regions of interest would behave and this would also avoid an artificial sample size increase. 

**Table 2:** Shapiro-Wilk test.

Tooth	Statistic	Sig.
#11	0.850	0.157
#12	0.902	0.389
#13	0.972	0.909
#21	0.954	0.775
#22	0.957	0.797
#23	0.831	0.110

## RESULTS

For the evaluation of the mean values of distances and root mean square (RMS) between the surfaces of the roots of T_1_ in relation to T_0_, they were compared to each other by means of colored maps. The descriptive statistics (maximum and minimum values, mean, standard-deviation, and one sample *t-*test) were calculated and are described in Table 1.

**Figure 4:**
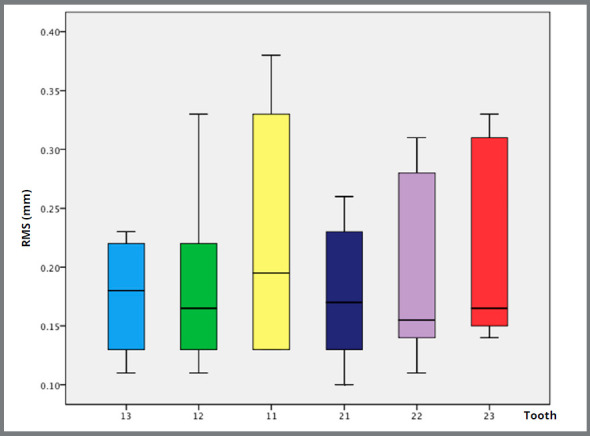
Distribution of the data of the RMS ( in mm ) values, exemplified by the box-plot: Maximum, minimum, and median measurements (vertical axis = RMS in mm; and horizontal axis = the corresponding tooth ).

The RMS value was used to evaluate the differences between the roots. The RMS, in fact, corresponds to the absolute mean of the distances in a normalized form, useful when there are large variations of values, both positive and negative.^19^ RMS for a collection of “n” values {X_1_, X_2_, ..., X_n_} is given by the formula below:

Considering the normal distribution of the sample, the one sample *t*-test was performed to evaluate if the values observed were statistically different from zero. As all values were above 0.05, no statistically significant differences between the mean and the desired error value were identified (Fig 4).

## DISCUSSION

The CBCT was used to evaluate the morphological changes caused by the retraction of the maxillary anterior teeth. The choice for this type of evaluation was based on the fact that there were no clinical studies in the literature evaluating the ERR by means of the superposition of 3D models, although some studies^8^ ^,^ ^20^ ^,^ ^21^ consider computed tomography (CT) as a better alternative in relation to radiographs. 

ERR is a common clinical complication of orthodontic treatment, being frequently seen by orthodontists, and is usually diagnosed in clinical practice by routine radiographs (panoramic or periapical), in which permanent reduction of the root tip of the tooth is observed. This type of resorption is generally asymptomatic and, when the loss of root structure by resorption becomes severe, the physiology and survival of the affected teeth can be compromised .^1^ ^,^ ^21^ In most cases, resorption resulting from orthodontic movement is minimal and has no clinical significance, reaching mean values of 0.5 to 3 mm of root shortening.^2^

CBCT is a powerful complementary diagnostic method to assess apical root resorption during orthodontic treatment, and it can be better than conventional radiograph, which underestimates root resorption.^21^ The introduction of CBCT technology can still be considered quite recent, and the literature still shows few research dedicated to studying its accuracy and specificity for the diagnosis of ERR. Due to the lack of evidences, the present study aimed to assess the root morphology of maxillary canines and incisors in patients submitted to four premolar extraction and orthodontic retraction of anterior teeth. The choice of these teeth in particular was due to the fact that the maxillary incisors are the teeth most frequently affected by root resorption. The degree of root resorption can be correlated to the magnitude of apex displacement and the length (treatment time) of the orthodontic treatment.^22^ Sameshima and Sinclair^23^ reported that root resorption mostly occurred in the anterior teeth rather than in the posterior teeth of the maxilla in 868 orthodontic patients. 

Even though two different space closure techniques were used in this study, previous paper has shown that there was no difference in the root resorption associated with *en masse* or two-stage closure.^24^ The present sample size does not allow any statement on this topic, but we also did not observe any difference between the two techniques. 

Although the CT is an imaging method superior to other radiographic methods for visualizing bone tissue, the accuracy of CT scanning in visualizing tooth root resorption is not well-known. Artifacts may affect the diagnostic reliability, such as beam-hardening effects, linear and nonlinear partial volume effects, edge gradient effects, and metal artifacts.^20^ Regarding the quality of CBCT, when root resorption can be identified in a CBCT scan with low resolution, it can mean that the resorption is present. However, if a CBCT scan did not show resorption in a highly suspicious case, then we can indicate a scan with high-resolution.^4^ Liedke et al.^25^ assessed the effect of CBCT resolution on the accuracy of root resorption measurements, and demonstrated that the CBCT approach was a reliable tool for assessing root resorption, and the 0.3-mm voxel resolution is the best configuration, because it associates great diagnostic performance with lower patient exposure to radiation.

Spatial resolution is the minimum distance required to distinguish two objects of similar density in a tomographic image, and may be incorrectly assumed to be equal to the scan’s reported resolution of a scan or voxel size. Spatial resolution defines the ability of the CBCT to separate two close objects, which can be improved by decreasing voxel size and increasing scan time. However, this can be detrimental due to increased radiation exposure and possible patient movement. Factors such as partial volume averaging, artifacts, and noise make it impossible to have a spatial resolution equivalent to the smallest voxel size.^26^ ^,^ ^27^

Spatial resolution and its contributing factors should be considered during the design or interpretation of CBCT studies. In the study of Ballrick et al,^26^ the authors found that a 0.2-mm voxel scan had an average spatial resolution of 0.4 mm. The two most common voxel sizes used for orthodontic scans, 0.3 and 0.4 mm - both averaged a spatial resolution of 0.7 mm -, should be used with caution if the goal is to assess small variations in bone thickness. In areas of thin bone, a spatial resolution of 0.7 mm would not be appropriate to visualize the bone, thus requiring a smaller voxel size, and would also decrease the influence of partial volume averaging.^26^

In this study, a tendency to light resorption was observed in the means of each tooth, since their values were negative, however very close to zero. Even when considering the extreme values, they did not exceed the spatial resolution measure, which in this sample was approximately 0.7mm. The lowest value was -0.7mm and the higher value was 0.13mm, that is, the extreme values are less than 0.7mm, demonstrating results consistent with the accuracy of the exam.

The *t*-test confirmed that the means observed for all teeth were not statistically different from zero, thus showing that there was no significant resorption. The small variability of the results, besides showing the absence of ERR in the evaluated sample, also evidences the consistency of the method used, showing that the accuracy of the same is within the limits of the exams’ spatial resolution.

Using helical computerized tomography, Fuhrmann^28^ verified that only bone plates with thickness of less than 0.2mm may not be visible in medical computed tomography. To date, no study has scored what would be the smallest thickness of bone plates that could be identified in the CBCT image. 

It can be said that the voxel dimension used to obtain the image is directly related to the dose of radiation to which the patient will be submitted during the procedure. Therefore, before selecting the image acquisition protocol, it is necessary to know its cost-benefit relationship, following the ALARA (As Low As Reasonably Achievable) principle. In other words, the professional should choose the exam protocol that presents the lowest radiation dose possible, but at the same time, is sufficiently sharp to identify the structures that need to be evaluated.^29^

There is no evidence that the detection of moderate to severe ERR differs between 2D and CBCT radiography or that its discovery during treatment leads to a different treatment decision in both techniques. However, identifying lingual or buccal root resorption could contribute to treatment decisions, as it would be detectable only by a CBCT.^30^

When compared to conventional CT scanners, CBCT machines are considered a less expensive and smaller equipment that exposes the patient to approximately 20% of the radiation of a helical CT, which is equivalent to the dose from a full-mouth periapical series.^31^ Even at the highest settings of the CBCT, the radiation dose is very below conservative limits recommended by the National Council on Radiation Protection and Measurements.^32^ There are two main reasons for using CBCT: in research, it increases our knowledge of root resorption; and in clinics, CBCT images may help to monitor the risk of developing root resorption during orthodontic tooth movement, in patients with agenesis or syndromes.^21^

Consideration of spatial resolution in orthodontic diagnosis is inexorable in the evaluation of small anatomical regions or when the objective is to identify small differences between the evaluated regions. Therefore, the evaluation of ERR is quite challenging, as well as the evaluation of bone plates.

## CONCLUSION

Based on the methodology applied and the results obtained, it is possible to present the following conclusion: There was no trend of ERR associated with orthodontic retraction of maxillary anterior teeth in the evaluated sample.
